# The PsyTAR dataset: From patients generated narratives to a corpus of adverse drug events and effectiveness of psychiatric medications

**DOI:** 10.1016/j.dib.2019.103838

**Published:** 2019-03-15

**Authors:** Maryam Zolnoori, Kin Wah Fung, Timothy B. Patrick, Paul Fontelo, Hadi Kharrazi, Anthony Faiola, Nilay D. Shah, Yi Shuan Shirley Wu, Christina E. Eldredge, Jake Luo, Mike Conway, Jiaxi Zhu, Soo Kyung Park, Kelly Xu, Hamideh Moayyed

**Affiliations:** a Lister Hill National Center for Biomedical Communications, National Library of Medicine, National Institutes of Health, Bethesda, MD, United States; b Department of Health Informatics & Administration, University of Wisconsin Milwaukee, Milwaukee, WI, United States; c Department of Health Sciences Research, Mayo Clinic, Rochester, MN, United States; d Department of Health Policy and Management, Johns Hopkins University, Baltimore, MD, United States; e Department of Biomedical and Health Information Sciences, University of Illinois at Chicago, Chicago, IL, United States; f School of Pharmacy, University of Pittsburgh, Pittsburgh, PA, United States; g School of Information, University of South Florida, Tampa, FL, United States; h Department of Biomedical Informatics, Utah University, Salt Lake City, UT, United States; i Emmes Corporation, Rockville, MD, United States; j Department of Epidemiology, Johns Hopkins University, Baltimore, MD, United States; k College of Letters and Science, University of Wisconsin Milwaukee, WI, United States

## Abstract

The “Psychiatric Treatment Adverse Reactions” (PsyTAR) dataset contains patients’ expression of effectiveness and adverse drug events associated with psychiatric medications. The PsyTAR was generated in four phases. In the first phase, a sample of 891 drugs reviews posted by patients on an online healthcare forum, “askapatient.com”, was collected for four psychiatric drugs: Zoloft, Lexapro, Cymbalta, and Effexor XR. For each drug review, patient demographic information, duration of treatment, and satisfaction with the drugs were reported. In the second phase, sentence classification, drug reviews were split to 6009 sentences, and each sentence was labeled for the presence of Adverse Drug Reaction (ADR), Withdrawal Symptoms (WDs), Sign/Symptoms/Illness (SSIs), Drug Indications (DIs), Drug Effectiveness (EF), Drug Infectiveness (INF), and Others (not applicable). In the third phases, entities including ADRs (4813 mentions), WDs (590 mentions), SSIs (1219 mentions), and DIs (792 mentions) were identified and extracted from the sentences. In the four phases, all the identified entities were mapped to the corresponding UMLS Metathesaurus concepts (916) and SNOMED CT concepts (755). In this phase, qualifiers representing severity and persistency of ADRs, WDs, SSIs, and DIs (e.g., mild, short term) were identified. All sentences and identified entities were linked to the original post using IDs (e.g., Zoloft.1, Effexor.29, Cymbalta.31). The PsyTAR dataset can be accessed via Online Supplement #1 under the CC BY 4.0 Data license. The updated versions of the dataset would also be accessible in https://sites.google.com/view/pharmacovigilanceinpsychiatry/home.

**Table:** Specifications table

Subject area	Psychiatric medications, Consumer Health Informatics, Medical Standard Vocabularies
More specific subject area	Consumer health posts, Machine Learning Systems, Text mining, Adverse drug events, SNOMED CT, UMLS
Type of data	Categorical, string, numeric variables, analyzed
How data was acquired	Using an Application Program Interface (API)
Data format	Comma Separated Values (CSV)
Experimental factors	Sample consists of 891 of drug review posts collected randomly from a healthcare forum “askapatint.com” for four psychiatric medications including Zoloft, Cymbalta, Effexor XR, and Cymbalta.
Experimental features	Factors measure pharmacological aspects of psychiatric medications.
Data source location	Data collected from an online healthcare forum called “askapatint.com”, United States
Data accessibility Related research article	Provided as online supplement Zolnoori, M., Fung, K. W., Patrick, T. B., Fontelo, et al. (2019). A systematic approach for developing a corpus of patient reported adverse drug events: A case study for SSRI and SNRI medications. *Journal of biomedical informatics, 90*, 103091.

**Table:** 

**Value of the data** • The PsyTAR dataset can be used as a benchmark to train and evaluate the performance of lexicon-based systems and machine learning algorithms to identify adverse drug events (ADEs) and measure drug effectiveness from online healthcare forums, particularly for psychiatric medications. • The PsyTAR dataset can be used to train machine learning systems (e.g. neural network) for normalizing medical concepts in online healthcare communities by extracting the semantic links among the layperson expressions of medical terms and medical standard vocabularies. • The PsyTAR dataset can be used to evaluate the association between different types of ADEs and patient satisfaction (attitude) toward psychiatric medications. • The PsyTAR dataset may also be used to facilitate the seamless exchange of information between patients' expressions of ADEs in personal health records (PHR) and electronic health records (EHRs) [1].

## 1 Data

**Fig. 1:**
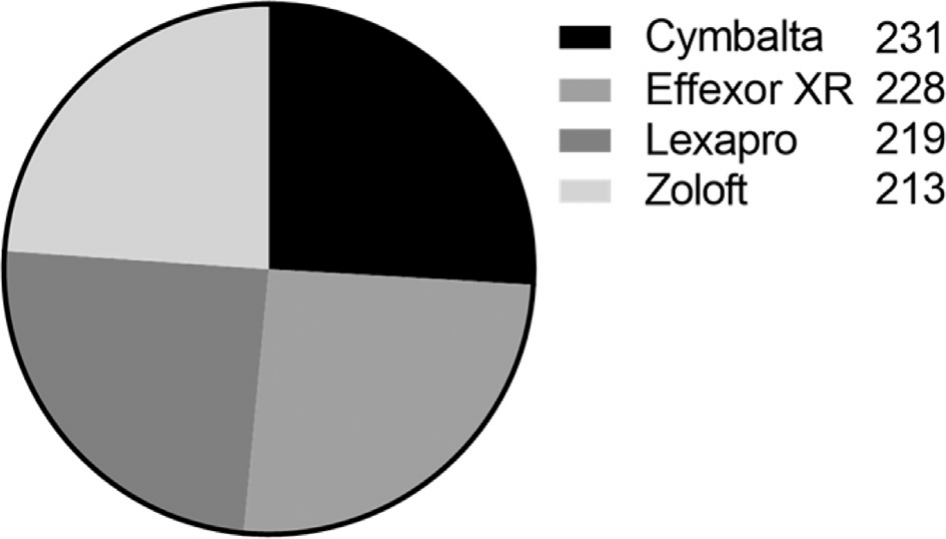
Sample sizes for the four drugs of the dataset.

**Fig. 2:**
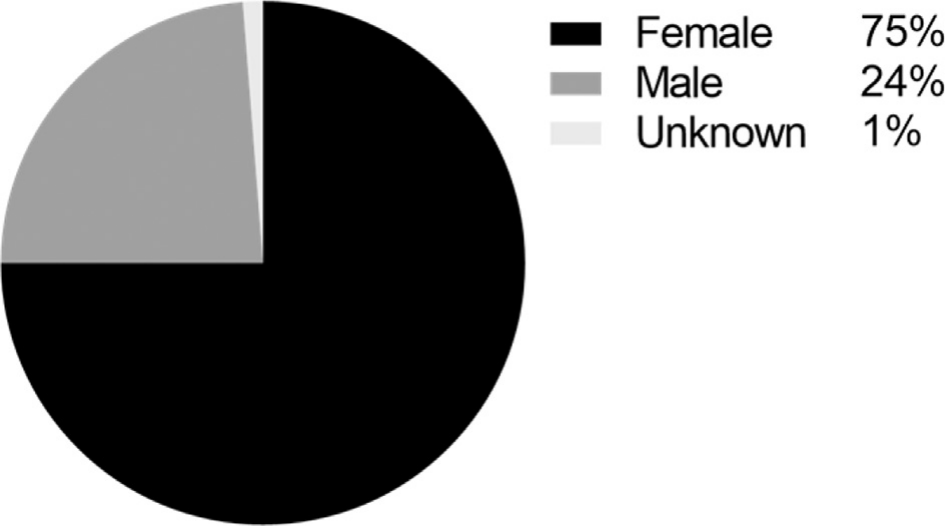
Gender distribution in the sample.

The sample of the PsyTAR contains 891 drug reviews collected randomly from an online healthcare forum “askapatient.com”. Fig. 1 shows the share of the sample for four drugs “Zoloft” and “Lexapro” from SSRIs (Selective Serotonin Reuptake Inhibitors) class and “Effexor XR” and “Cymbalta” from the SNRIs (Serotonin-Norepinephrine Reuptake Inhibitors) class. Fig. 2 shows the gender demographic distribution of the sample. The average of age and duration of usage were 37 and 18 months for the whole sample respectively.

**Fig. 3:**
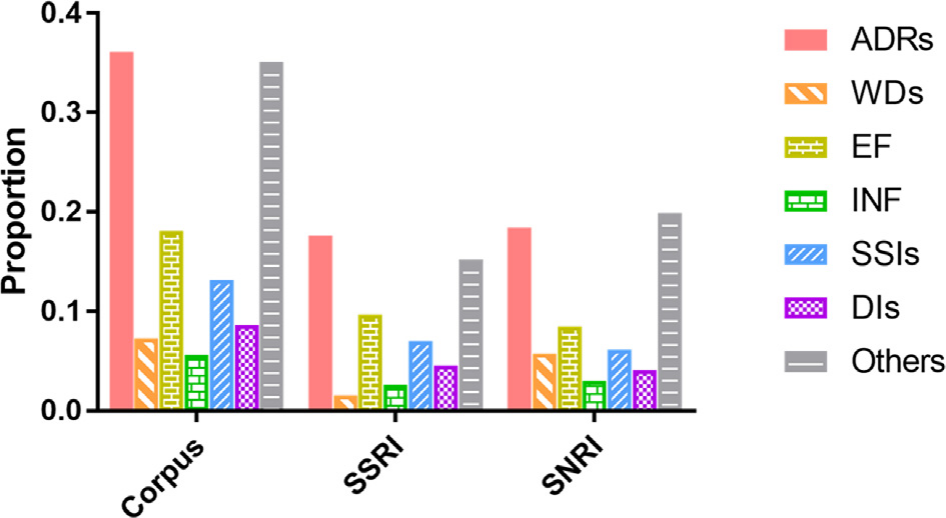
Frequency of sentences labeled for each item in the dataset, and SSRIs and SNRIs class separately.

In the second phase, drug review posts were split into sentences, and then sentences were labeled for the presence of ADRs (Adverse drug reaction), WDs (Withdrawal Symptoms), SSIs (sign, symptom, illness), DIs (Drug Indications), EF (drug effectiveness), and INF (drug ineffectiveness). The total number of sentences in the sample is 6009. Fig. 3 shows frequency of sentences labeled for each of these items for the whole PsyTAR dataset and SSRI and SNRI classes separately.

**Fig. 4:**
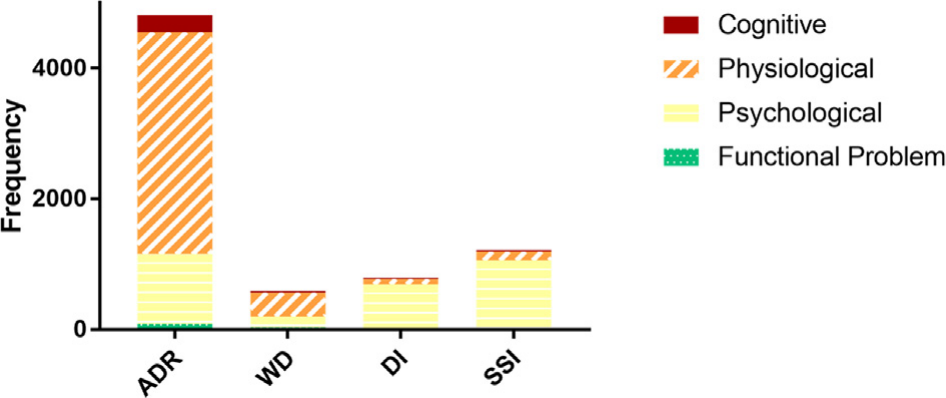
Frequency of cognitive, physiological, psychological, and functional problems entity type by ADRs, WDs, DIs, and SSIs for the entire dataset.

**Fig. 5:**
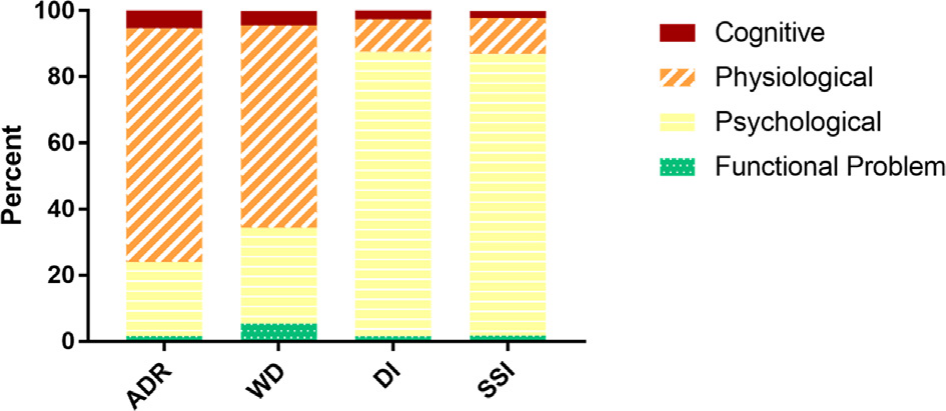
Percentage of cognitive, physiological, psychological, and functional problems entity types by ADRs, WDs, DIs, and SSIs in the entire dataset.

In the third phase, mentions of ADRs, WDs, SSIs, and DIs were identified and extracted from the sentences, and then classified as physiological, psychological, cognitive, or functional problem. Fig. 4 shows the total frequency of identified ADRs, WDs, DIs, and SSIs broken down by the type of entity including physiological, psychological, cognitive, and functional problems. Fig. 5 shows the percentage of identified ADRs, WDs, DIs, and SSIs for the entire PsyTAR dataset and type of entities separately.

**Fig. 6:**
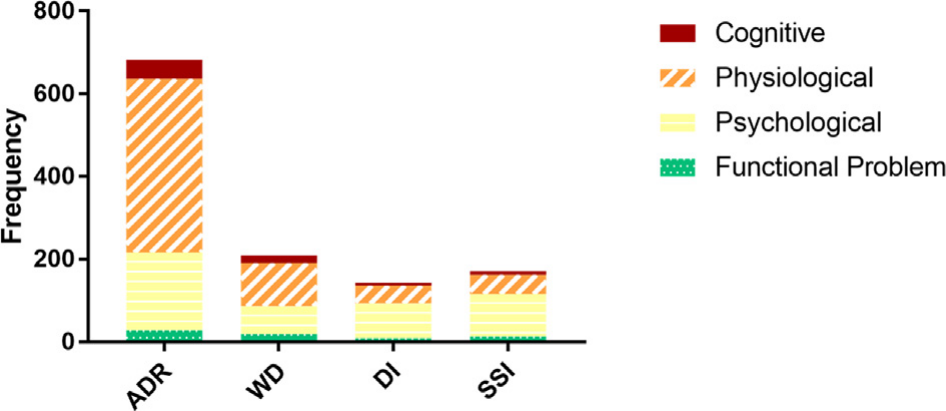
Frequency of UMLS concepts for each ADRs, WDs, DIs, SSIs after normalization.

**Fig. 7:**
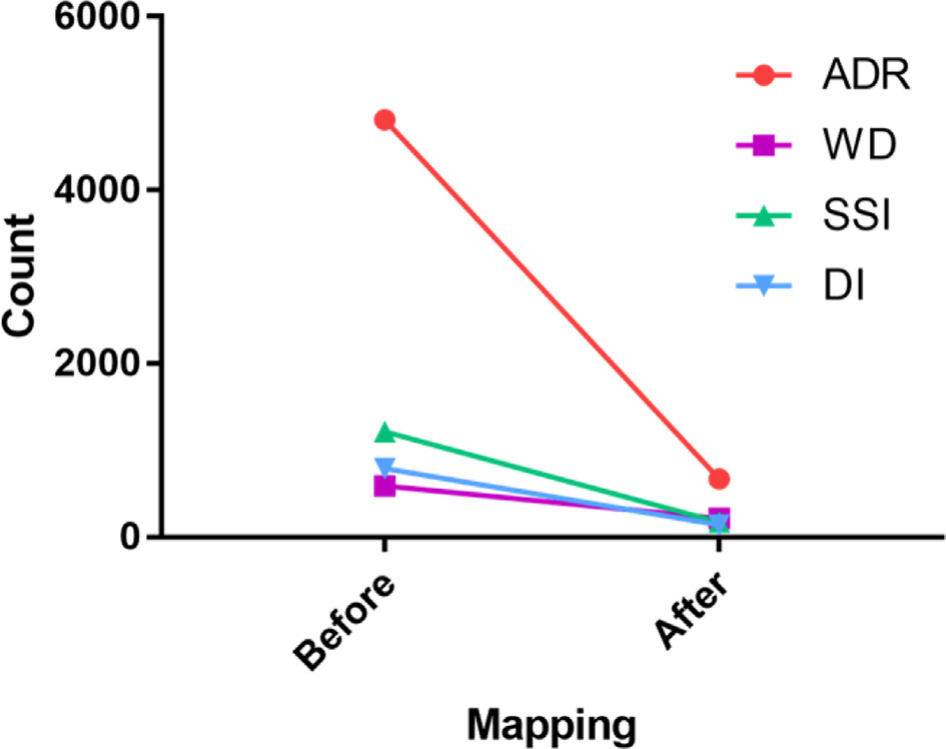
Reduction of identified entities by mapping to the UMLS Metathesaurus concepts.

In the fourth phase, all the identified entities were mapped to 918 unique UMLS concepts and 755 unique SNOMED CT concepts. Fig. 6 shows frequency of UMLS concepts for each ADRs, WDs, DIs, and SSIs. The 3180 unique identified ADRs in the third phase were mapped to 673 UMLS concepts, indicating the high semantic variabilities of patients expression of ADRs [1]. Fig. 7 shows the reduction of identified entities by mapping to the UMLS Metathesaurus concepts.

**Fig. 8:**
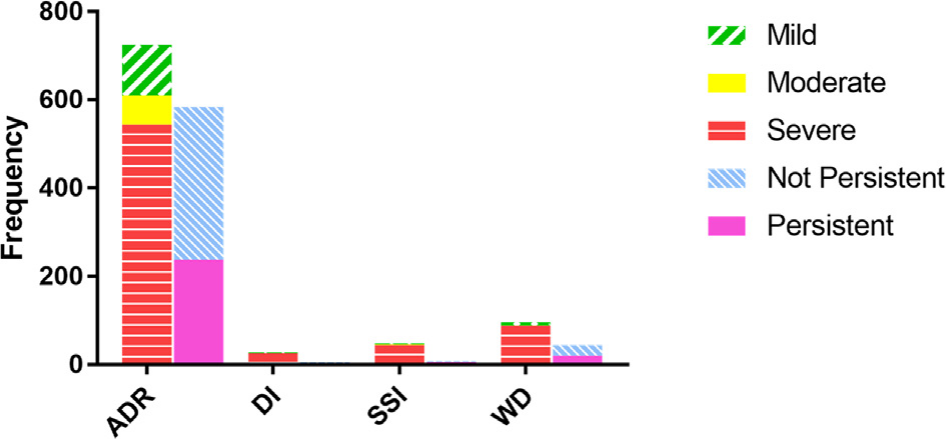
Frequency of identified entities indicating severity and persistency of the identified entities (ADR, WD, DI, SSI).

In this phase, we also identified qualifiers indicating severity and persistency of identified entities. Fig. 8 shows the frequency of identified qualifiers including “mild”, “moderate”, and “severe” indicating severity, and “persistent” and “not-persistent” indicating persistency of the identified entities (ADRs, WDs, DIs, SSIs).

## 2 Experimental design, materials and methods

The drug reviews were collected from a healthcare forum called “askapatient.com”. We developed an Application Programming Interface (API) to collect data from this forum. The sample size was calculated using the formula of sample size for qualitative studies [2]. In the next step, the drug reviews were processed for correcting grammatical errors and removing personal information (e.g., website, emails). Then, the reviews were split into sentences, and each sentence was double coded (labeled) for the presence of ADR, WD, DI, SSI, EF, and INF. The calculated inter-annotator agreement (IAA) using Kappa was 78% for the entire dataset. In the next phase, mentions of the ADR, WD, SSIs, and DIs were identified from the relevant sentences. Four annotators identified the boundary of the entities by strictly following guidelines developed for the entity identification phase. The calculated IAA for entity identification was 86% for the entire dataset. In the last phase, the identified entities were mapped to the corresponding UMLS Metathesaurus concepts and SNOMED CT concepts. All of the identified concepts were reviewed for consistency. The detailed methodology for developing this dataset is discussed in a separate manuscript [1].
