# Supplementary material for: The PsyTAR dataset: From patients generated narratives to a corpus of adverse drug events and effectiveness of psychiatric medications
Source: Data Brief. 2019 Mar 15;24:103838. doi: 10.1016/j.dib.2019.103838 (PMC6495095; doi:10.1016/j.dib.2019.103838)
Supplement: Multimedia component 1 [file mmc1.docx]

**Conflict of Interest**

We have no conflict of interest to declare.
